# Author reply to “Caution about overdiagnosis of neck calcification”

**DOI:** 10.1002/jgf2.406

**Published:** 2020-11-19

**Authors:** Tetsuya Akaishi, Tadashi Ishii

**Affiliations:** ^1^ Department of Education and Support for Regional Medicine Tohoku University Hospital Sendai Japan

To the Editor,

We thank the authors of the Letter to the Editor^1^ for their constructive and educational comments on our article, entitled “Retropharyngeal calcific tendinitis: A rare, benign, but painful condition with a stiff neck”.^2^ We agree with the importance of not overlooking cases of life‐threatening conditions, which may be hastily diagnosed as benign based on neck calcification. To further emphasize the importance, we revised the original Figure 2, as Figure 1 shown below.

**FIGURE 1:**
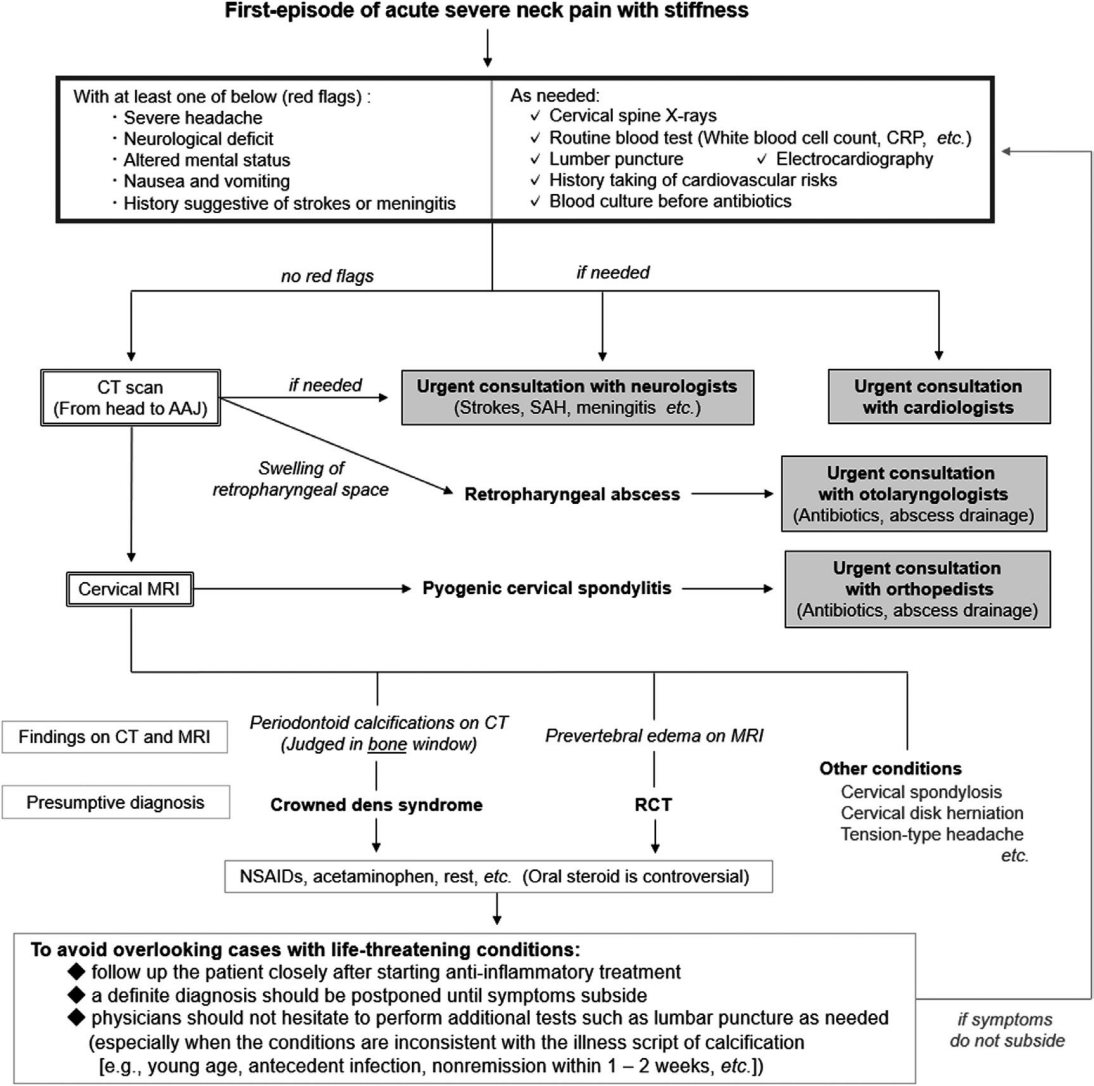
A revised version of the conceivable diagnostic strategy for patients with acute painful stiff neck. Abbreviations: AAJ, atlantoaxial joint; CRP, C‐reactive protein; NSAIDs, non‐steroidal anti‐inflammatory drugs; RCT, retropharyngeal calcific tendinitis; SAH, subarachnoid hemorrhage

Certainly, as the authors pointed out, following up on patients who do not present any red flags during the initial hospital visit is essential until the symptoms subside, because some patients with life‐threatening conditions, such as cerebral hemorrhage and meningitis, may initially lack them, especially older adults or patients suffering from cognitive impairment.^3^ Up to 30% of patients with pleocytosis may not show meningeal signs.^4^ Headaches may also be absent in approximately 10% of patients with bacterial meningitis.^5^ In cases of emergent conditions, a delayed diagnosis without a timely therapeutic intervention may cause severe irreversible neurological sequelae. On these premises, the comment from the authors to follow‐up on patients with presumptive diagnoses of benign conditions based on neck calcification until the symptoms subside is justified.

Therefore, we recommend that physicians should consult neurologists for possible additional diagnostic tests, such as lumbar puncture, for patients who were initially diagnosed with benign conditions based on neck calcification if they do not show the expected outcomes following anti‐inflammatory treatment. As the authors suggested, careful follow‐up of the patients is essential until the symptoms subside and emergent conditions are ruled out.

## CONFLICT OF INTEREST

The authors declare no conflicts of interest.
